# Unperturbed hydrocarbon chains and liquid phase bilayer lipid chains: a computer simulation study

**DOI:** 10.1007/s00249-017-1231-9

**Published:** 2017-07-11

**Authors:** Alexander L. Rabinovich, Alexander P. Lyubartsev, Dmitrii V. Zhurkin

**Affiliations:** 1 Institute of Biology, Karelian Research Center, Russian Academy of Sciences, Pushkinskaya 11, Petrozavodsk, 185910 Russian Federation; 2 Department of Materials and Environmental Chemistry, Stockholm University, 106 91 Stockholm, Sweden; 3 Physics and Technology Department, Petrozavodsk State University, Universitetskaya 10, Petrozavodsk, 185910 Russian Federation

**Keywords:** Lipid bilayers, Biomembranes, Unsaturated hydrocarbon chains, Molecular dynamics, Monte Carlo

## Abstract

In this work, the properties of saturated and unsaturated fatty acid acyl chains 16:0, 18:0, 18:1(n-9)*cis*, 18:2(n-6)*cis*, 18:3(n-3)*cis*, 18:4(n-3)*cis*, 18:5(n-3)*cis*, 20:4(n-6)*cis*, 20:5(n-3)*cis* and 22:6(n-3)*cis* in a bilayer liquid crystalline state and similar hydrocarbon chains (with CH$_3$ terminal groups instead of C=O groups) in the unperturbed state characterised by a lack of long-range interaction were investigated. The unperturbed hydrocarbon chains were modelled by Monte Carlo simulations at temperature $T = 303$ K; sixteen fully hydrated homogeneous liquid crystalline phosphatidylcholine bilayers containing these chains were studied by molecular dynamics simulations at the same temperature. To eliminate effects of the simulation parameters, the molecular dynamics and Monte Carlo simulations were carried out using the same structural data and force field coefficients. From these computer simulations, the average distances between terminal carbon atoms of the chains (end-to-end distances) were calculated and compared. The trends in the end-to-end distances obtained for the unperturbed chains were found to be qualitatively similar to those obtained for the same lipid chains in the bilayers. So, for understanding of a number of processes in biological membranes (e.g., changes in fatty acid composition caused by environmental changes such as temperature and pressure), it is possible to use, at least as a first approximation, the relationships between the structure and properties for unperturbed or isolated hydrocarbon chains.

## Introduction

Biological membranes are very complex heterogeneous systems composed of various molecules such as lipids, sterols, proteins, carbohydrates, etc. Lipid molecules contain different head groups and a wide variety of acyl chains of fatty acids (FAs; Cook and McMaster 2002; Nelson and Cox 2008; Mouritsen and Bagatolli 2016). The FAs are the fundamental building blocks of all lipids in living matter. The most abundant class of lipids in the biological membranes of animals and plants is phosphatidylcholine (PC). FA acyl chains of PC lipids usually contain 12–24 carbon atoms; the most common chain lengths fall between 14 (or 16) and 22. Most of the FA acyl chains are unsaturated, containing 1–6 double bonds of the *cis* configuration in different positions; the majority of the double bonds in the tails are methylene-interrupted (i.e., one methylene group is localized between each pair of double bonds; Cook and McMaster 2002; Nelson and Cox 2008; Mouritsen and Bagatolli 2016). It is most common to find chains with an even number of carbon atoms, whereas odd ones are found in rare cases.

The FA chains are often denoted in accordance with ‘n-minus’ nomenclature, as $N:d(n-j)cis$, where *N* refers to the total number of carbon atoms in the chain, *d* is the number of the methylene-interrupted double bonds and *j* is the position of the first double bond, counted from the methyl (CH$_3$) terminus of the chain, with the methyl carbon as number 1. For brevity, below the term $(n-j)cis$ in the notation will be occasionally omitted.

It should be mentioned that unsaturated FA chains and especially polyunsaturated (PU) tails of lipids (for instance, 18:1(n-9)*cis* FA, 18:2(n-6)*cis* FA, 18:3(n-3)*cis* FA, 18:4(n-3)*cis* FA, 18:5(n-3)*cis* FA, 20:4(n-6)*cis* FA, 20:5(n-3)*cis* FA and 22:6(n-3)*cis* FA) are of great importance for the structure and function of animal and plant membranes (Table 1).

**Table 1:** Occurrence of unsaturated and polyunsaturated (PU) fatty acid (FA) chains of lipids in animal and plant membranes

Fatty acid chain	Occurrence (findings)	Refs.
18:1(n-9)*cis* FA	The most abundant monoenoic FA in plant and animal tissues	
	35–60% of the total FAs of peanut oil acylglycerols	Carrin and Carelli (2010)
	35–69% of the total FAs of peanut oil acylglycerols	Köckritz and Martin (2008)
	60% of the total FAs of the oil from 00-quality oilseed rape	Wittkop et al. (2009)
	40–70% of various vegetable oils	Pinzi et al. (2009)
	91–92% of HO sunflower 90plus oil	Köckritz and Martin (2008)
	30–40% of the total FAs in adipose fats of animals	Nelson and Cox (2008)
18:2(n-6)*cis* FA	An ubiquitous component of plant lipids	
	~58% in the cold-pressed black cumin seed oil	Lutterodt et al. (2010)
	48–74% of sunflower oil	Köckritz and Martin (2008)
	48–59% of soybean oil	Köckritz and Martin (2008)
	47–58% of cottonseed oil	Köckritz and Martin (2008)
	75% of the total FAs of heart cardiolipin of animals	Minkler and Hoppel (2010)
18:3(n-3)*cis* FA	Large amounts in flaxseed and walnuts	
	34% of the total FAs in garden cress seed oil	Diwakar et al. (2010)
	56–71% of the total FAs in linseed oil	Köckritz and Martin (2008)
18:4(n-3)*cis* FA	Variable amounts in several species of fungi and animals tissues, in seeds of some plant families	Guil-Guerrero (2007)
	Up to 27% in several species of algae	Guil-Guerrero (2007)
	Up to 18% in *Echium* (Boraginaceae) species	Guil-Guerrero (2007)
18:5(n-3)*cis* FA	Certain algal groups in marine phytoplankton	Napolitano et al. (1995)
20:4(n-6)*cis* FA	The mammalian cell membranes, fish oils, etc.	
	30–70% of the total FAs of triacylglycerols produced by the filamentous fungus *Mortierella alpina* 1S-4	Sakuradani (2010)
20:5(n-3)*cis* FA	One of the most important FAs of the so-called ‘(n-3) family’: animal tissues (especially brain), algae, fish oils	DHA (2009)
22:6(n-3)*cis* FA	The most unsaturated FA commonly found in nature, it regulates many cell transport and synaptic functions	
	A major constituent of fish oils, especially from tuna eyeballs	DHA (2009)
	40% of the PU FAs in the brain of animals	DHA (2009)
	60% of the PU FAs in the retina of animals	DHA (2009)

It should also be noted that all higher plants have the ability to synthesize 18:2(n-6)*cis* FA and 18:3(n-3)*cis* FA, and some can also synthesize 18:4(n-3)*cis* FA (Singh et al. 2005). The chains of 18:2(n-6)*cis*, 18:3(n-3)*cis* and 18:4(n-3)*cis* FAs accumulate in plant tissues as terminal FA metabolites (Rincón-Cervera and Guil-Guerrero 2010). 22:6(n-3)*cis* FA is usually the end point of 18:3(n-3)*cis* FA metabolism in animal tissues. On the whole, membranes that are active metabolically, as in rod outer segments, mitochondria, synaptic vesicles, etc., have high levels of PU chains.

**Fig. 1:**
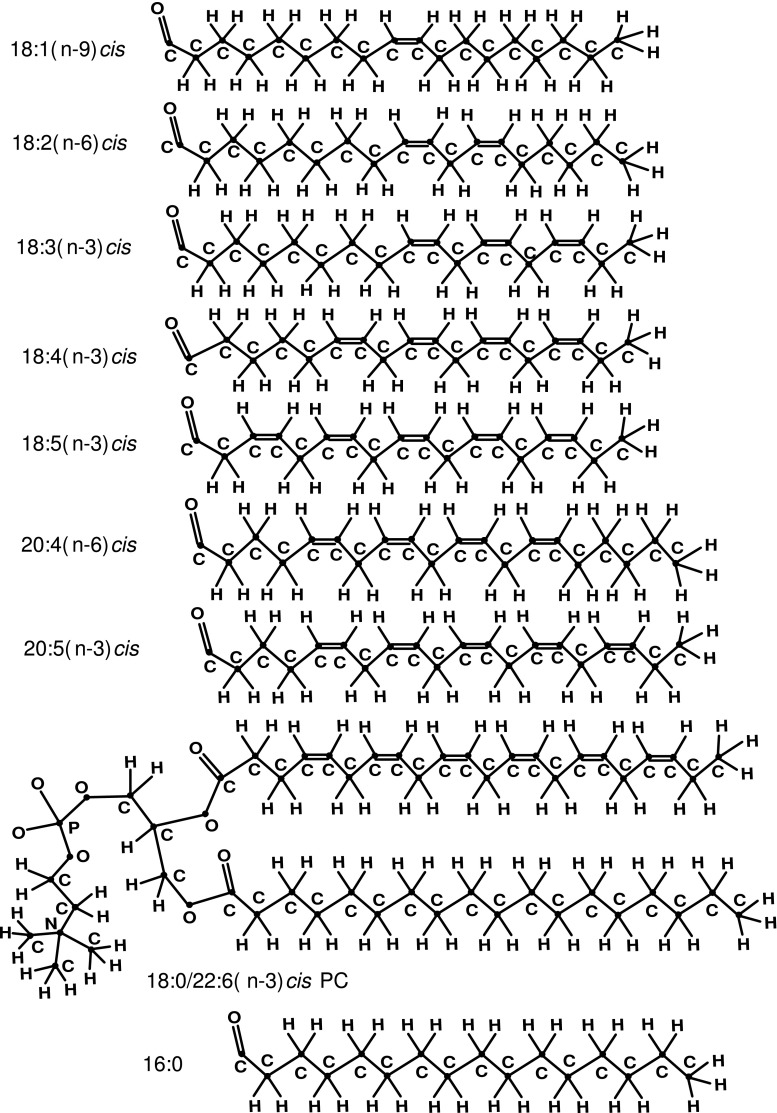
Structures, from top to bottom, of *sn*-2 lipid chains (fatty acid acyls) of 18:1(n-9)*cis*, 18:2(n-6)*cis*, 18:3(n-3)*cis*, 18:4(n-3)*cis*, 18:5(n-3)*cis*, 20:4(n-6)*cis* and 20:5(n-3)*cis*; phosphatidylcholine molecule of 18:0/22:6(n-3)*cis* PC showing structures of *sn*-2 22:6(n-3)*cis* acyl chain and *sn*-1 18:0 acyl chain; structure of the possible *sn*-1 16:0 acyl chain

Chemical structures of all above-mentioned *sn*-2 FA acyls and one of the PC molecules [18:0/22:6(n-3)*cis* PC molecule] are presented in Fig. 1.

PU FA chains have been linked to a great number of biochemical processes. One of the most notable, observations is that PU FAs play a role in achieving optimal health and in protection against disease. In other words, PU FAs (and their derivatives) have significant clinical implications. The beneficial health effects of PU FAs, particularly, 22:6(n-3)*cis* FA and 20:5(n-3)*cis* FA (DHA 2009; Sahena et al. 2009) are related to several tens of human afflictions, such as cancer (Nabavi et al. 2015; D’Eliseo and Velotti 2016; Molfino et al. 2016), cardiovascular disease (Rovere and Christensen 2015; Sperling and Nelson 2016), allergic diseases (Rueter et al. 2015), many skin disorders (McCusker and Grant-Kels 2010), and diabetes (Bhaswant et al. 2015; Wang and Chan 2015), etc.

Thus, it is very important to study physical properties of lipid acyl chains in different conditions to reveal relationships between chemical structure and physical properties. Indeed, such relationships are of great importance for understanding the structure and functioning of biomembranes.

Unfortunately, experimental data for different properties of such hydrocarbons or FA acyl chains are scarce or lacking. Computer simulation is nowadays one of the most powerful tools for studying the properties of different molecular systems (Leach 2001; Berendsen 2007; Gould et al. 2007; Landau and Binder 2009; Binder and Heermann 2010; Brooks et al. 2011; Satoh 2011) including lipid membranes, lipids and lipid chains (see, e.g., several reviews on MD simulations of lipid membranes: Bennett and Tieleman 2013; Rabinovich and Lyubartsev 2013; Pluhackova and Böckmann 2015; Baoukina and Tieleman 2016; Bunker et al. 2016; Kirsch and Böckmann 2016; Lyubartsev and Rabinovich 2016; Pasenkiewicz-Gierula et al. 2016; Pöyry and Vattulainen 2016, and other articles published in Special Issue 10 of BBA-Biomembranes, 2016, v.1858 entitled: Biosimulations) because it allows one to obtain information on an atomistic level. On the other hand, computer simulations of lipid bilayer systems with all possible combinations of chains are still very time-consuming, and, therefore, a different approach to the task would be valuable.

To obtain ‘structure–property’ relationships for different hydrocarbon chains which can be compared with each other, one should use uniform conditions: the same state at the same temperature. The ‘unperturbed’ state of chain molecules (Flory 1969) was used as the uniform state of hydrocarbon chains in this work. The exact definition of this state is presented below, in the ‘2.2 Monte Carlo simulations’ section. It has been proposed (Flory 1969) that properties of chain molecules in this state correspond to the properties in the bulk amorphous state. Neutron scattering experiments were later carried out (Dettenmaier 1978; Yoon and Flory 1978) and the results substantiated this prediction. On the other hand, biological membranes in a physiological form exist in a liquid crystalline (fluid) state having a relatively high degree of disorder and dynamical behaviour; this state is vital for the proper functioning of membranes.

The aim of the present study was to compare properties of a set of hydrocarbon chains in the unperturbed state unaffected by long-range interactions and for comparison in the liquid crystalline state of lipid bilayers, to assess if these properties are similar to each other.

A structural scheme of the chains considered in the present work is as follows: CH$$_3$$–(CH$$_2)_a$$–(CH=CH–CH$$_2)_d$$–(CH$$_2)_b$$–CH$$_3$$,

where *a*, *d*, *b* are integers. The total number of carbons of the chain is $N = a + 3d + b +2$. For clear visualization of the connection between structure of the chains and FA acyl chains, these hydrocarbons will be denoted below as $alk-N:d(n-j)cis$, i.e., similar to the ‘n-minus’ nomenclature for FAs.

**Fig. 2:**
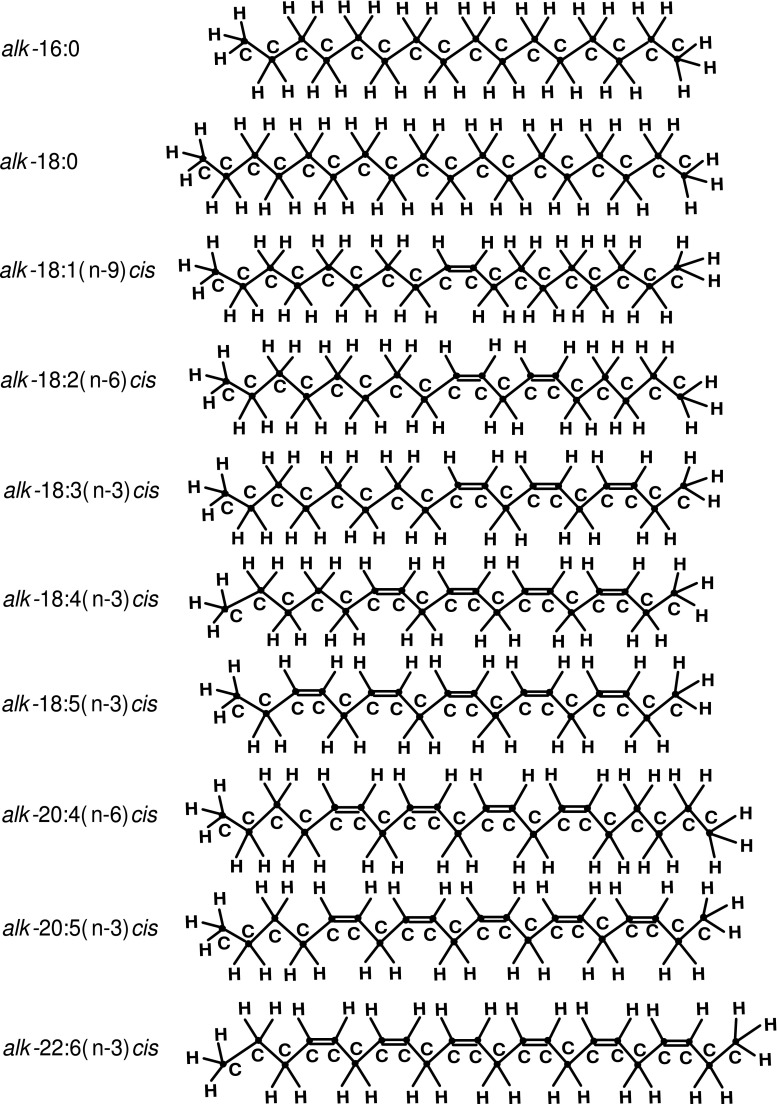
Structures, from top to bottom, of hydrocarbon chains *alk*-16:0, *alk*-18:0, *alk*-18:1(n-9)*cis*, *alk*-18:2(n-6)*cis*, *alk*-18:3(n-3)*cis*, *alk*-18:4(n-3)*cis*, *alk*-18:5(n-3)*cis*, *alk*-20:4(n-6)*cis*, *alk*-20:5(n-3)*cis* and *alk*-22:6(n-3)*cis* studied by Monte Carlo simulations. Such names of the hydrocarbon chains are used to stress the chain and corresponding FA acyl (Fig. 1) structural similarity

The chains of *alk*-16:0, *alk*-18:0, *alk*-18:1(n-9)*cis*, *alk*-18:2(n-6)*cis*, *alk*-18:3(n-3)*cis*, *alk*-18:4(n-3)*cis*, *alk*-18:5(n-3)*cis*, *alk*-20:4(n-6)*cis*, *alk*-20:5(n-3)*cis* and *alk*-22:6(n-3)*cis* were studied by Monte Carlo (MC) simulations in an unperturbed state at temperature $T = 303$ K. Chemical structures of the 10 studied hydrocarbon chains are presented in Figure 2.

In addition, 16 fully hydrated homogeneous liquid crystalline PC bilayers containing these chains (as FA acyls) were studied by molecular dynamics (MD) simulations at the same temperature ($T = 303$ K). The MD and MC simulations were carried out using the same force field, to eliminate the effect of the simulation parameters. Both techniques are described below.

## Models and methods

### Molecular dynamics simulations

Sixteen fully hydrated homogeneous phosphatidylcholine (PC) bilayers were studied by MD simulation in an NPT-ensemble at temperature $T=303$ K and pressure $P=1$ bar. The simulation software was the MDynaMix package (Lyubartsev and Laaksonen 2000). The bilayers studied were comprised of one of the PC molecules which contained a saturated *sn*-1 chain (16:0 or 18:0) and an unsaturated *sn*-2 chain:

1-palmitoyl-2-oleoyl-*sn*-glycero-3-PC [16:0/18:1(n-9)*cis* PC];

1-stearoyl-2-oleoyl-*sn*-glycero-3-PC [18:0/18:1(n-9)*cis* PC];

1-palmitoyl-2-linoleoyl-*sn*-glycero-3-PC [16:0/18:2(n-6)*cis* PC];

1-stearoyl-2-linoleoyl-*sn*-glycero-3-PC [18:0/18:2(n-6)*cis* PC];

1-palmitoyl-2-linolenoyl-*sn*-glycero-3-PC [16:0/18:3(n-3)*cis* PC];

1-stearoyl-2-linolenoyl-*sn*-glycero-3-PC [18:0/18:3(n-3)*cis* PC];

1-palmitoyl-2-octadecatetraenoyl-*sn*-glycero-3-PC [16:0/18:4(n-3)*cis* PC];

1-stearoyl-2-octadecatetraenoyl-*sn*-glycero-3-PC [18:0/18:4(n-3)*cis* PC];

1-palmitoyl-2-octadecapentaenoyl-*sn*-glycero-3-PC [16:0/18:5(n-3)*cis* PC];

1-stearoyl-2-octadecapentaenoyl-*sn*-glycero-3-PC [18:0/18:5(n-3)*cis* PC];

1-palmitoyl-2-arahidonoyl-*sn*-glycero-3-PC [16:0/20:4(n-6)*cis* PC];

1-stearoyl-2-arahidonoyl-*sn*-glycero-3-PC [18:0/20:4(n-6)*cis* PC];

1-palmitoyl-2-eicosapentaenoyl-*sn*-glycero-3-PC [16:0/20:5(n-3)*cis* PC];

1-stearoyl-2-eicosapentaenoyl-*sn*-glycero-3-PC [18:0/20:5(n-3)*cis* PC];

1-palmitoyl-2-docosahexaenoyl-*sn*-glycero-3-PC [16:0/22:6(n-3)*cis* PC];

1-stearoyl-2-docosahexaenoyl-*sn*-glycero-3-PC [18:0/22:6(n-3)*cis* PC].

The choice of the lipid set was motivated by the following considerations: (1) the sixteen bilayers listed contain the most important, biologically meaningful types of PU lipids (see “Introduction” section); (2) An inspection of these bilayers under the same conditions allows one to study:

1. the ‘double bond number dependence’ (‘*d* dependence’) of an *sn*-2 chain of lipid properties over the whole possible range of *d* from 1 to 5 at fixed $N = 18$ in a sequence of 18:1, 18:2, 18:3, 18:4 and 18:5 chains with methylene-interrupted double bonds, all other factors being equal;
2. the effect of *sn*-1 chain elongation (from 16 to 18 carbons) on the lipid properties for different fixed *sn*-2 chains, all other factors being equal;
3. the effect of *sn*-2 chain elongation (from 18 to 20 carbons) on the lipid properties for different fixed *sn*-1 chains and fixed double bond number $d = 4$ or 5 of *sn*-2 chain, all other factors being equal.

The presence of the acyl chain 16:0 in the 8 bilayers 16:0/...PC and acyl chain 18:0 in the 8 bilayers 18:0/...PC is sufficient to study geometrical properties of acyls 16:0 and 18:0 by MD simulation. End-to-end distances (between carbons) calculated for each saturated acyl were averaged over eight corresponding PC bilayers; the influence of the position (*sn*-1 instead of *sn*-2) of these chains on their end-to-end distances was neglected.

Experimental melting temperatures $T_c$ of the bilayers were checked to make sure that the temperature $T=303$ K in the MD simulations was appropriate. Available published experimental temperatures $T_c$ for the lamellar gel to liquid-crystalline phase transition of five of eight studied mixed-chain PCs with 16:0 chains in the *sn*-1 position are gathered in Tables 2 and 3, and six of eight simulated PCs with 18:0 *sn*-1-chains are gathered in Tables 4 and 5. To the authors’ knowledge, no experimental investigations of the melting temperatures of 16:0/18:4(n-3)*cis* PC, 16:0/18:5(n-3)*cis* PC, 16:0/20:5(n-3)*cis* PC, 18:0/18:4(n-3)*cis* PC or 18:0/18:5(n-3)*cis* PC have been published.

**Table 2:** Published temperatures $T_c$ of the experimental lamellar gel to liquid-crystalline phase transition of phosphatidylcholines 16:0/18:1(n-9)*cis* PC

Lipid	$T_c$ (*K*)	Method	Refs.
16:0/18:1(n-9)*cis* PC	266.15^a^	Raman spectr.	Lavialle and Levin (1980)
	268.15	DSC	Kruyff et al. (1973)
	268.15	^2^H NMR	Waespe-Sarcevic (1978
			Perly et al. (1985) and Ghosh (1988)
	268.15	SANS	Winter and Pilgrim (1989)
	268.55 ± 0.07^b^	DSC	Tada et al. (2009)
	268.55	DSC	Tada et al. (2010)
	268.65	DSC	Santaren et al. (1982)
	270.15	DSC	Davis et al. (1980) and Dekker et al. (1983)
			Curatolo et al. (1985)
			Lynch and Steponkus (1989)
	270.55 ± 0.2	DSC	Davis et al. (1981) and Keough (1986)
	270.65	DSC	Swaney (1980)
	270.65^a^	Raman spectr.	Lavialle and Levin (1980)
	270.65	Raman spectr.	Litman et al. (1991)
	270.65 ± 0.2	DSC	Hernandez-Borrell and Keough (1993)
	271.15	DSC	Curatolo (1985) and Curatolo (1986)
	271.55	DSC	Ichimori et al. (1999)
	271.65^c^	Raman spectr.	Lavialle and Levin (1980)
	272.95 ± 0.77	DSC	Bryant et al. (1992)
	276.15	Hydrol. meas.	Kamp et al. (1975)
	270.65 ± 2.4	lipid database	Koynova and Caffrey (1998)

*DSC* differential scanning calorimetry, $^2$ *H NMR* deuterium nuclear magnetic resonance, *SANS* small-angle neutron scattering, *Hydrol. meas.* hydrolysis measure. $T_c$ is the temperature averaged over the gel to liquid-crystalline and liquid-crystalline to gel phase transition temperatures, i.e., the heating and cooling transition temperatures, in all cases when a hysteresis was observed. The $T_c$ values for each lipid are presented in order of increasing temperature; values from the lipid database (Koynova and Caffrey 1998) are also presented in the end of the experimental data list.

^a^The existence of metastable forms for the pure 16:0/18:1(n-9)*cis* PC liposomes was detected in Ref. Lavialle and Levin (1980). The ${I_{2940}}$/${I_{2885}}$ peak height intensity ratio as index was used (interchain disorder-order parameter), where $I_{2940}$ and $I_{2885}$ represent the peak height intensities for 2940- and 2885-$cm^{-1}$ transitions.

^b^Aqueous 50 wt% ethylene glycol solution.

^c^The $I_{1100}$/$I_{1130}$ peak height intensity ratio as index was used (intramolecular gauche-trans isomerization parameter), where $I_{1100}$ and $I_{1130}$ represent the peak height intensities for 1100- and 1130-$cm^{-1}$ transitions, respectively

**Table 3:** Published temperatures $T_c$ of the experimental lamellar gel to liquid-crystalline phase transition of mixed-chain phosphatidylcholines containing an *sn*-1 palmitoyl (16:0) chain and various *sn*-2 unsaturated fatty acid chains

Lipid	$T_c$ (*K*)	Method	Refs.
16:0/18:2(n-6)*cis* PC	253.15	DSC	Lynch and Steponkus (1989)
	253.65	DSC	Keough et al. (1989)
	254.45	DSC	Hernandez-Borrell and Keough (1993)
	$253.55\pm 0.4$	Lipid database	Koynova and Caffrey (1998)
16:0/18:3(n-3)*cis* PC	$258.15\pm 0.5$	$^2$H NMR	McCabe et al. (1994)
16:0/20:4(n-6)*cis* PC	250.65	Raman spectr.	Litman et al. (1991)
	$252.55\pm 0.2$	DSC	Hernandez-Borrell and Keough (1993)
	250.65	Lipid database	Koynova and Caffrey (1998)
16:0/22:6(n-3)*cis* PC	261.85	DSC	Hernandez-Borrell and Keough (1993)
	$265.3\pm 0.5$	$^2$H NMR	Barry et al. (1991)
	266.65	$^2$H NMR	Deese et al. (1981)
	270.15	Raman spectr.	Litman et al. (1991)
	270.15	Lipid database	Koynova and Caffrey (1998)

For abbreviations, see footnote in Table 2

**Table 4:** Published temperatures $T_c$ of the experimental lamellar gel to liquid-crystalline phase transition of phosphatidylcholines 16:0/18:1(n-9)*cis* PC

Lipid	$T_c$ (*K*)	Method	Refs.
18:0/18:1(n-9)*cis* PC	$272.0\pm 0.2$	DSC	Niebylski and Jr. (1994)
	275.15	Fluor. anis.	Vincent et al. (1985)
	276.15	DSC	Phillips et al. (1972)
	$277.25\pm 0.1$	DSC	Sánchez-Migallón et al. (1996)
	$277.95\pm 0.5$	$^2$H NMR	Holte et al. (1995)
	$277.95\pm 0.08$ ^a^	DSC	Tada et al. (2009)
	278.20	DSC	Lüscher-Mattli (1989)
	278.45	DSC	Cunningham et al. (1989)
	278.75	DSC	Wang et al. (1995b, 1995a) and Huang et al. (1996)
	278.85	DSC	Inoue et al. (1999)
	279.05	DSC	Stillwell et al. (2000)
	279.15	DSC	Vilchéze et al. (1996)
	279.35	DSC	Surewicz and Epand (1986)
	$279.45\pm 0.4$	DSC	Davis et al. (1980, 1981) and Keough (1986)
	279.55	DSC	Davis et al. (1986)
	279.75	DSC	Boggs and Tümmler (1993)
	279.85	DSC	Kaneshina et al. (1998) and Ichimori et al. (1999)
			Broniec et al. (2009) and Tada et al. (2010)
	$279.85\pm 0.1$ ^b^	DSC	Tada et al. (2009)
	280.55	DSC	Davis and Keough (1983)
	281.35	DSC	Dai et al. (1991)
	286.15	Hydrol. meas.	Kamp et al. (1975)
	$280.05\pm 2.9$	Lipid database	Koynova and Caffrey (1998)

For abbreviations, see footnote in Table 2; fluor. anis. = fluorescence anisotropy

^a^ Aqueous 50 wt% ethylene glycol solution

^b^ Solvent: water

**Table 5:** Published temperatures $T_c$ of the experimental lamellar gel to liquid-crystalline phase transition of mixed-chain phosphatidylcholines containing an *sn*-1 stearoyl (18:0) chain and various *sn*-2 unsaturated fatty acid chains

Lipid	$T_c$ (*K*)	Method	Ref.
18:0/18:2(n-6)*cis* PC	$256.45\pm 0.3$	DSC	Sánchez-Migallón et al. (1996)
	256.65	DSC	Coolbear and Keough (1983)
	$256.95\pm 1.6$	DSC	Coolbear et al. (1983)
	$257.95\pm 0.1$	DSC	Niebylski and Jr. (1994)
	$257.95\pm 0.5$	$^2$H NMR	Holte et al. (1995)
	258.15	DSC	Keough and Parsons (1990) and Tada et al. (2010)
	259.45	DSC	Keough et al. (1989)
	$258.75\pm 4.1$	Lipid database	Koynova and Caffrey (1998)
18:0/18:3(n-3)*cis* PC	$259.25\pm 0.2$	DSC	Sánchez-Migallón et al. (1996)
	260.15	DSC	Coolbear et al. (1983)
	$260.25\pm 0.3$	DSC	Niebylski and Jr. (1994)
	261.15	DSC	Coolbear and Keough (1983)
	$261.25\pm 0.5$	$^2$H NMR	Holte et al. (1995)
	$260.85\pm 1.2$	Lipid database	Koynova and Caffrey (1998)
18:0/20:4(n-6)*cis* PC	$257.55\pm 0.3$	DSC	Sánchez-Migallón et al. (1996)
	$258.45\pm 1.0$	DSC	Niebylski and Jr. (1994)
	$259.65\pm 0.5$	$^2$H NMR	Holte et al. (1995)
	260.15	DSC	Ichimori et al. (1999) and Tada et al. (2010)
	$260.55\pm 1.0$	DSC	Coolbear et al. (1983)
	$260.25\pm 0.4$	Lipid database	Koynova and Caffrey (1998)
18:0/20:5(n-3)*cis* PC	$260.55\pm 0.5$	$^2$H NMR	Holte et al. (1995)
	$262.05\pm 0.1$	DSC	Niebylski and Jr. (1994)
	$262.75\pm 0.1$	Lipid database	Koynova and Caffrey (1998)
18:0/22:6(n-3)*cis* PC	263.95	DSC	Dumaual et al. (2000) and Stillwell et al. (2000)
	264.15	DSC	Tada et al. (2010)
	$265.60\pm 0.5$	$^2$H NMR	Holte et al. (1995)
	265.95	DSC	Ichimori et al. (1999)
	$266.55\pm 0.3$	DSC	Niebylski and Jr. (1994)
	$266.65\pm 0.7$	$^2$H NMR	Barry et al. (1991)
	$269.35\pm 1.8$	Lipid database	Koynova and Caffrey (1998)

For abbreviations, see footnote in Table 2

It is seen that the temperature $T = 303$ K of the MD computer simulations is higher than experimental gel to liquid-crystalline phase transition temperatures $T_c$ of all the bilayers in Tables 2, 3, 4 and 5. In spite of the fact that $T_c$ values of several lipids, 16:0/18:4(n-3)*cis* PC, 16:0/18:5(n-3)*cis* PC, 16:0/20:5(n-3)*cis* PC, 18:0/18:4(n-3)*cis* PC and 16:0/18:5(n-3)*cis* PC, are unknown, there is good reason to believe from the analysis of the noted Tables that missing values of $T_c$ are also less than $T = 303$ K. This temperature is acceptable also for MC simulations of hydrocarbon chains because the main phase transition temperature of octadecane (*alk*-18:0) is 301.2 *K* and that of hexadecane (*alk*-16:0) is 291.2 *K* (Dirand et al. 2002), and phase transition temperatures of unsaturated (alkene) chains are substantially lower than those of n-alkanes.

A description of the MD simulations technique of PC bilayers was presented in a previous paper (Rabinovich and Lyubartsev 2014). The simulation boxes contained 128 PC molecules of one of 16 studied types per bilayer (64 lipids in each leaflet) and 30 H$_2$O molecules per lipid that corresponds to a condition of full hydration (overall 3840 water molecules). The two hydrocarbon tails, the glycerol section and the head group of the lipid molecules were treated in accordance with their known chemical structure, all hydrogen atoms were explicitly included in the computations.

In the starting configuration, the lipids were set parallel to each other, organized in a regular manner in two layers, and water molecules were distributed outside the bilayer. The system was put into a rectangular periodic cell, with the *Z* axis parallel to the bilayer normal. The size of the box was varied during the simulations under a semianisotropic NPT-ensemble with two degrees of freedom: one in the *Z* direction and another in the *XY* direction, so that the box sizes in *X* and *Y* direction were equal at each time moment.

To calculate the energy of the lipid molecules in the course of MD simulations, the CHARMM27 force field parameter set (Feller and MacKerell, Jr. 2000) with modifications described in a previous paper (Högberg et al. 2008) was used. It was demonstrated in a number of publications that the original CHARMM27 force field has some disagreements with the experiment (Benz et al. 2005), especially in the tensionless isothermal-isobaric (NPT) ensemble simulations (Hyvönen and Kovanen 2005; Sonne et al. 2007). CHARMM27 force field was recommended to apply only with a fixed surface area; otherwise the simulated bilayer tends to form a gel-like state (Feller et al. 2002; Koubi et al. 2003; Jensen et al. 2004; Siu et al. 2008). For bilayers composed of 14:0/14:0 PC lipids, modifications introduced previously (Högberg et al. 2008) provided perfect agreement with experimental data for the area per lipid, as well as with the X-ray structure factor and NMR order parameters. For lipids considered in this work, we used the same partial charges as in Högberg et al. 2008, the lipid head group including esters (these charges were recalculated on the bases of ab-initio computations), while for tails, we used charges adopted from the original CHARMM27 force field (Feller and MacKerell, Jr. 2000), with scaling of 1–4 electrostatic interactions by factor 0.83 (Högberg et al. 2008). All intramolecular bond and angle parameters, as well as Lennard-Jones interactions, were also borrowed from the original CHARMM27 force field (Feller and MacKerell, Jr. 2000). Water molecules were described by the flexible SPC model (Toukan and Rahman 1985). Use of this water model, in connection with the modified CHARMM27 force field, was verified previously (Högberg et al. 2008).

The double time step algorithm (Tuckerman et al. 1992) was used to treat separately fast forces (covalent bonds, angles, torsions, collision Lennard-Jones forces within 5 Å distance) with time step 0.25 fs, and longer range forces with time step 2.5 fs. The long-range electrostatic interactions were treated by the Ewald summation method (Allen and Tildesley 1987). The reciprocal part of the Ewald sum was cut at the condition that the remaining terms do not contribute more than 0.0001 of the total value. The $\alpha$ parameter of the Ewald sum was set to $\alpha =2.6 r_{\rm cut}$, and cut-off distance $r_{\rm cut}=14$ Å was optimized for computational performance according to Ref. (Fincham 1994). The dispersion correction from the Lennard-Jones interactions outside the cut-off distance was included in the pressure (Allen and Tildesley 1987).

The systems were firstly simulated 1 ns under constant volume and then 1 ns under constant pressure and isotropic cell fluctuations. The obtained configurations were considered as starting points for longer simulations with independent cell fluctuations in Z and XY directions. The time reversible Nose-Hoover constant-temperature–constant-pressure algorithm (Martyna et al. 1996) was implemented, with the thermostat and barostat relaxation time set to 30 fs and 1 ps, respectively.

**Fig. 3:**
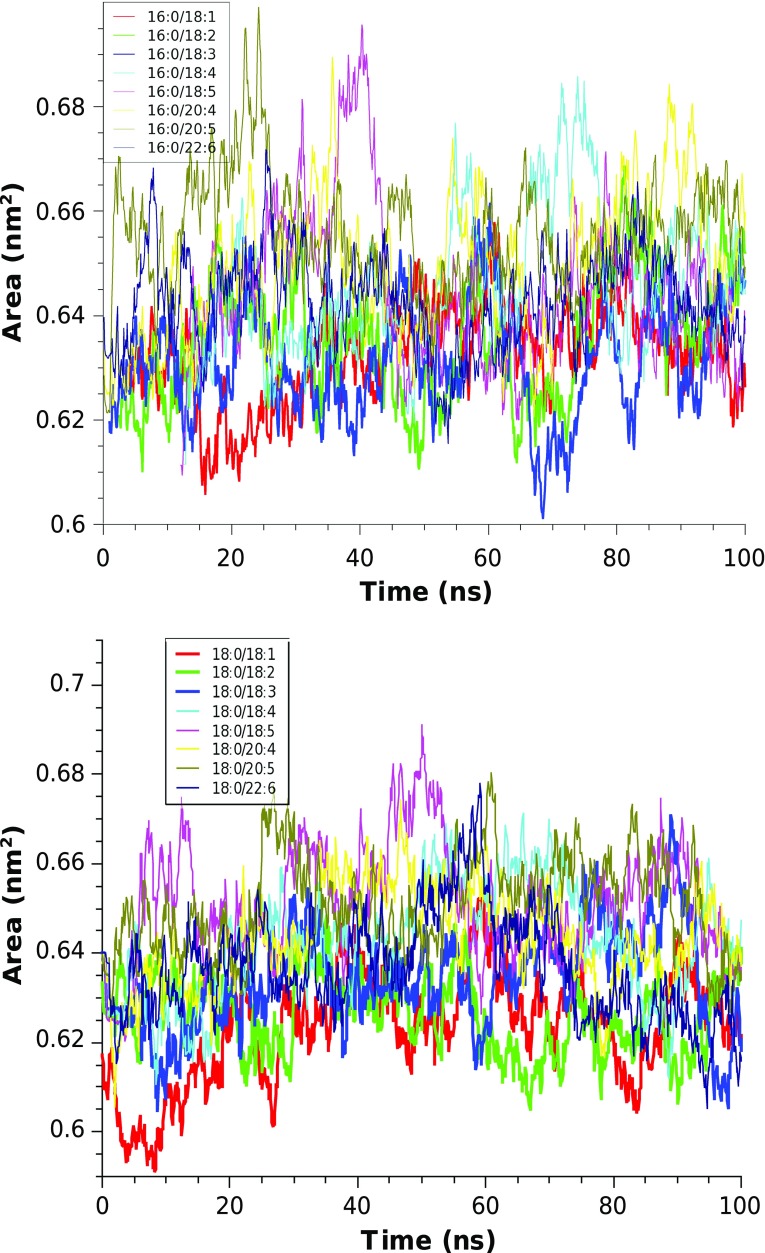
Time evolution of the average area per lipid $A_{\rm pl}$ of the PC bilayers with *sn*-1 chain 16:0 (a) and 18:0 (b)

All the PC bilayers were simulated for a total of 100 ns. One of the most fundamental properties of a lipid bilayer and one of the most common ways to determine whether the bilayer system has reached equilibrium is area per lipid $A_{\rm pl}$. When the area per lipid reaches a stable value, other structural properties (density distributions, NMR order parameters) do not show noticeable trends either. In the present work, area per lipid $A_{\rm pl}$ was calculated as the cross-sectional area of the simulation box divided by the number of lipids per monolayer. The time evolution of the $A_{\rm pl}$ values of the each of 16 PC bilayers are shown in Figs. 3a, b.

From the observation of the different time evolution traces and calculation of block averages, we concluded that 20 ns of equilibration time is enough for all the bilayer systems considered. Therefore, the first 20 ns of the simulations (from 100 ns) were disregarded in further analysis. Atomic coordinates were saved each 1 ps in the trajectories.

The average areas $A_{\rm pl}$ calculated as a result of our simulations are presented in Table 6.

**Table 6:** Average areas per lipid, $A_{\rm pl}$, and relative fluctuations of the areas obtained for mixed-chain liquid-crystalline phase unsaturated phosphatidylcholine bilayers by MD simulations of the present work; $T = 303$ K

Lipid	$A_{\rm pl}$ ($\pm$ $\Delta$)^a^, $nm^2$	Rel. fluct. of area^a^
16:0/18:1(n-9)*cis* PC	0.634 (±0.002)	0.01436
16:0/18:2(n-6)*cis* PC	0.636 (±0.003)	0.01839
16:0/18:3(n-3)*cis* PC	0.632 (±0.002)	0.01835
16:0/18:4(n-3)*cis* PC	0.647 (±0.003)	0.02087
16:0/18:5(n-3)*cis* PC	0.646 (±0.004)	0.02369
16:0/20:4(n-6)*cis* PC	0.652 (±0.003)	0.01932
16:0/20:5(n-3)*cis* PC	0.656 (±0.003)	0.01602
16:0/22:6(n-3)*cis* PC	0.643 (±0.002)	0.01446
18:0/18:1(n-9)*cis* PC	0.627 (±0.002)	0.01515
18:0/18:2(n-6)*cis* PC	0.625 (±0.002)	0.01503
18:0/18:3(n-3)*cis* PC	0.637 (±0.002)	0.01790
18:0/18:4(n-3)*cis* PC	0.646 (±0.003)	0.01702
18:0/18:5(n-3)*cis* PC	0.653 (±0.003)	0.01915
18:0/20:4(n-6)*cis* PC	0.646 (±0.003)	0.01516
18:0/20:5(n-3)*cis* PC	0.653 (±0.002)	0.01685
18:0/22:6(n-3)*cis* PC	0.637 (±0.004)	0.02024

^a^ Statistical error $\Delta$ for 20–100 ns is evaluated from the variance of 10-ns block averages

^b^ $$(\langle (A_{\rm pl} - \langle A_{\rm pl} \rangle )^2\rangle )^{1/2}$$/$$\langle A_{\rm pl} \rangle$$

It is possible to compare our data with the available published (experimental) data: in Table 7, available experimental average lipid areas for bilayers formed by PC lipids with one fully saturated and one unsaturated chain are collected. It is seen that our results are in good agreement with the experimentally deduced data.

**Table 7:** Published experimental average areas per lipid, $A_{\rm pl}$, of liquid-crystalline phase mixed-chain PC bilayers (^a^estimated from a plot)

Lipid	*T* (K)	$A_{\rm pl}$ ($nm^2$)	Method	Refs.
16:0/18:1(n-9)*cis* PC	275	$0.54\pm 0.01$	X-ray scattering	Pabst et al. (2000)
	293	$0.627\pm 0.013$	X-ray scattering	Kučerka et al. (2011)
	297	0.63	Langmuir film balance	Smaby et al. (1997)
	298	$0.64\pm 0.01$	Isopiestic method	Klose et al. (1992)
				Köenig et al. (1997a)
	298	$0.65\pm 0.03$	Fluoresc. reson. energy transf.	Lantzsch et al. (1994) and Lantzsch et al. (1996)
	298	$0.66\pm 0.02$	X-ray diffraction	König (1992)
				Lantzsch et al. (1996)
	301	$0.593\pm 0.012$	$^2$H NMR	Leftin et al. (2014)
	301	$0.604\pm 0.036$	$^{13}$C NMR	Leftin et al. (2014)
	303	$0.643\pm 0.013$	X-ray scattering	Kučerka et al. (2011)
	303	$0.683\pm 0.015$	X-ray scattering	Kučerka et al. (2005)
	310	0.66	Surface-pressure measur.	Hyslop et al. (1990)
	310	$0.668\pm 0.005$	Small-angle X-ray diffraction	Jerabek et al. (2010)
	321	$0.662\pm 0.013$	$^2$H NMR	Leftin et al. (2014)
	321	$0.705\pm 0.042$	$^{13}$C NMR	Leftin et al. (2014)
	323	$0.62\pm 0.01$	X-ray scattering	Pabst et al. (2000)
	323	$0.673\pm 0.013$	X-ray scattering	Kučerka et al. (2011)
16:0/18:2(n-6)*cis* PC	297	0.66	Langmuir film balance	Smaby et al. (1997)
16:0/20:4(n-6)*cis* PC	297	0.68	Langmuir film balance	Smaby et al. (1997)
16:0/22:6(n-3)*cis* PC	297	0.70	Langmuir film balance	Smaby et al. (1997)
18:0/18:1(n-9)*cis* PC	293	$0.638\pm 0.013$	X-ray scattering	Kučerka et al. (2011)
	303	$0.614\pm 0.006$	$^2$H NMR and X-ray	Köenig et al. (1997b)
	303	0.643	‘Compressibility’ method	Rand and Parsegian (1989)
	303	$0.655\pm 0.013$	X-ray scattering	Kučerka et al. (2011)
	303	0.66	Osmotic pressure technique	Rand et al. (1988)
	303	0.66	Gravimetric method	Rand and Parsegian (1989)
	303	0.666	$^2$H and $^{31}$P NMR	Separovich and Gawrisch (1996)
				Gawrisch and Holte (1996)
	303	0.67^a^	Low-angle X-ray scattering	Pan et al. (2009)
	303	0.71^a^	Wide-angle X-ray scattering	Pan et al. (2009)
	323	0.681±0.014	X-ray scattering	Kučerka et al. (2011)
18:0/18:2(n-6)*cis* PC	303	0.673	$^2$H and $^{31}$P NMR	Separovich and Gawrisch (1996)
				Gawrisch and Holte (1996)
18:0/18:3(n-3)*cis* PC	303	0.666	$^2$H and $^{31}$P NMR	Separovich and Gawrisch (1996)
				Gawrisch and Holte (1996)
18:0/20:4(n-6)*cis* PC	303	0.706	$^2$H and $^{31}$P NMR	Separovich and Gawrisch (1996)
				Gawrisch and Holte (1996)
18:0/20:5(n-3)*cis* PC	303	0.691	$^2$H and $^{31}$P NMR	Separovich and Gawrisch (1996)
				Gawrisch and Holte (1996)
18:0/22:6(n-3)*cis* PC	303	$0.682\pm 0.004$	X-ray diffraction	Eldho et al. (2003)
	303	$0.692\pm 0.009$	$^2$H NMR and X-ray	Köenig et al. (1997b)
	303	0.716	$^2$H and $^{31}$P NMR	Separovich and Gawrisch (1996)
				Gawrisch and Holte (1996)

### Monte Carlo simulations

According to concepts developed by Flory (1969), the interpretation of the spatial configuration of a linear chain molecule dispersed in a dilute solution can be resolved into two parts: short-range and long-range interactions. (1) The short-range interactions of the chain are determined by the geometrical parameters (bond lengths and bond angles), together with the potentials affecting rotation about bonds, including the effects of steric interactions between atoms and groups which are near neighbors in sequence along the chain; in other words, the short-range effects are determined by interactions between groups separated by only a few bonds. (2) The long-range interactions are dominated by interactions involving pairs of atoms and groups which are remote in the chain sequence, though near to one another in space when involved in mutual interactions; to put it differently, the long-range interactions are determined by interactions between pairs which are separated by many bonds (Flory 1969).

The long-range interactions introduce alterations (perturbation) in the chain configuration obtained when only the short-range interactions are considered. It is important to note that the long-range effect depends not only on the actual volume of the chain group (fragment, unit) but also on its interaction with the solvent; it is reasonable, therefore, to discuss the effective covolume. The covolume for the chain group can be enhanced by use of a ‘good’ solvent for the chain. It may also be diminished by choice of a ‘poor’ one barely capable of dissolving the chain. Through judicious selection of solvent and temperature, the finite volume of the group can be compensated exactly by the mutual attractions between chain groups when immersed in the poor solvent (Flory 1969). This state was called the ‘Theta ($\Theta$) point’; as this takes place, the perturbation of the chain configuration must vanish and the chain become unperturbed (Flory 1969). Further, according to the prediction by Flory, in the bulk amorphous state, perturbation of the chain configuration must vanish. Neutron scattering experiments were later carried out (Dettenmaier 1978; Yoon and Flory 1978), and the results substantiated this prediction.

The ‘unperturbed’ state of chain molecules was used as the uniform state of the different hydrocarbon chains in this work. From the mathematical point of view, to reach this state, the long-range interactions should be excluded.

Thus, the MC simulations were performed here for unperturbed hydrocarbon chains, in which only intramolecular interactions between near neighbours along the chain were included. Let *U* be the conformational energy of a chain in the unperturbed state, i.e., the short-range interaction energy. The equilibrium properties of all chains were calculated using the classical flexible model (Gö and Scheraga 1976). The average value of the physically observable $\langle H \rangle _{\Theta }$ of an unperturbed molecule in the canonical ensemble for this model is given by

1 $$\begin{aligned} \langle H \rangle _{\Theta } = \frac{\int \limits _0^{2\pi }\ldots \int \limits _0^{2\pi } H(\varphi _1,\ldots , \varphi _{N - 1}) \cdot \exp [ - U(\varphi _1,\ldots , \varphi _{N - 1}) / k_BT] d \varphi _1 \ldots d \varphi _{N - 1}}{\int \limits _0^{2\pi }\ldots \int \limits _0^{2\pi } \exp [ - U(\varphi _1,\ldots , \varphi _{N - 1} ) / k_BT] d\varphi _1 \ldots d \varphi _{N - 1}}, \end{aligned}$$

where *N* is the number of carbons of the main chain; $k_B$—the Boltzmann constant; *T*—temperature; and $\varphi _1, \varphi _2, \ldots , \varphi _{N - 1}$ are torsion angles of the main chain. Here, in (1) and below in the MC procedure, we keep bond lengths and bond angles equal to their equilibrium values which correspond to the chosen force field parameters; the torsions for all double bonds arranged *cis* were also fixed at the equilibrium value.

Assume we generate angles $\varphi _1,\varphi _2,\ldots , \varphi _{N-1}$ with probability density $p(\varphi _1,\varphi _2,\ldots , \varphi _{N-1})$. Then, an assessment $\overline{H}_{\omega }$ of the value $\langle H \rangle _{\Theta }$ by the MC method (Gould et al. 2007; Landau and Binder 2009; Binder and Heermann 2010; Satoh 2011) is

2 $$\begin{aligned} \overline{H}_{\omega } = \frac{\sum \nolimits _{\nu = 1}^\omega H( \varphi _1^\nu ,\ldots , \varphi _{N-1}^\nu ) \cdot \exp [ - U (\varphi _1^\nu ,\ldots , \varphi _{N-1}^\nu ) / k_B T] / p( \varphi _1^\nu ,\ldots , \varphi _{N-1}^\nu ) }{\sum \nolimits _{\nu = 1}^\omega \exp [ - U( \varphi _1^\nu ,\ldots , \varphi _{N-1}^\nu ) / k_BT] / p( \varphi _1^\nu ,\ldots , \varphi _{N-1}^\nu )}. \end{aligned}$$

Here, $\omega$ is the sample size and $\nu$ is the number of the current conformation. The value $\overline{H}_{\omega }$ from expression (2) converges to the value $\langle H \rangle _{\Theta }$ from expression (1).

In previous work (Zhurkin and Rabinovich 2015), an important sampling technique was developed for the efficient generation of chain conformations, with continuous variation of all single C–C bond torsions within [0, $2\pi$] range. The conformations were generated using the probability density

3 $$\begin{aligned} p(\varphi _1,\ldots , \varphi _{N-1}) \approx \frac{ \exp [ - U(\varphi _1,\ldots , \varphi _{N - 1}) / k_BT]}{\int \limits _0^{2\pi }\ldots \int \limits _0^{2\pi } \exp [ - U(\varphi _1,\ldots , \varphi _{N - 1} ) / k_BT] d\varphi _1 \ldots d \varphi _{N - 1}}. \end{aligned}$$

To calculate the conformational energy *U* of a hydrocarbon chain in the unperturbed state (the short-range interactions energy), a scheme of interdependence of each of three torsions along the chain was taken into account in our work. The energy *U* was calculated as the sum of energies $U_m (\varphi _{\gamma }, \varphi _{\gamma + 1}, \varphi _{\gamma + 2})$ of $N_f$ structural units (e.g., $N_f$ = $N-3$ for a saturated chain with *N* carbons):

4 $$\begin{aligned} U = \sum \limits _{\gamma = 1}^{N_f} {U_{m_{\gamma }} (\varphi _{\gamma }, \varphi _{\gamma + 1}, \varphi _{\gamma + 2})}. \end{aligned}$$

where $\varphi _{\gamma }, \varphi _{\gamma + 1}, \varphi _{\gamma + 2}$ are torsion angles, and *m* is the structural unit type. The units reproduced precisely the structure of various chain fragments.

The energy *U* is arranged [according to (4)] in such a way that it is possible to calculate energy $U_{m_{\gamma }}$ of any *m* unit at the preliminary step, before MC simulation of the chain. To calculate the energies of all units and chains as a whole, the same force field parameters as in the MD simulations were used (CHARMM27; Feller and MacKerell, Jr. 2000) with modifications performed in the paper (Högberg et al. 2008).

**Fig. 4:**
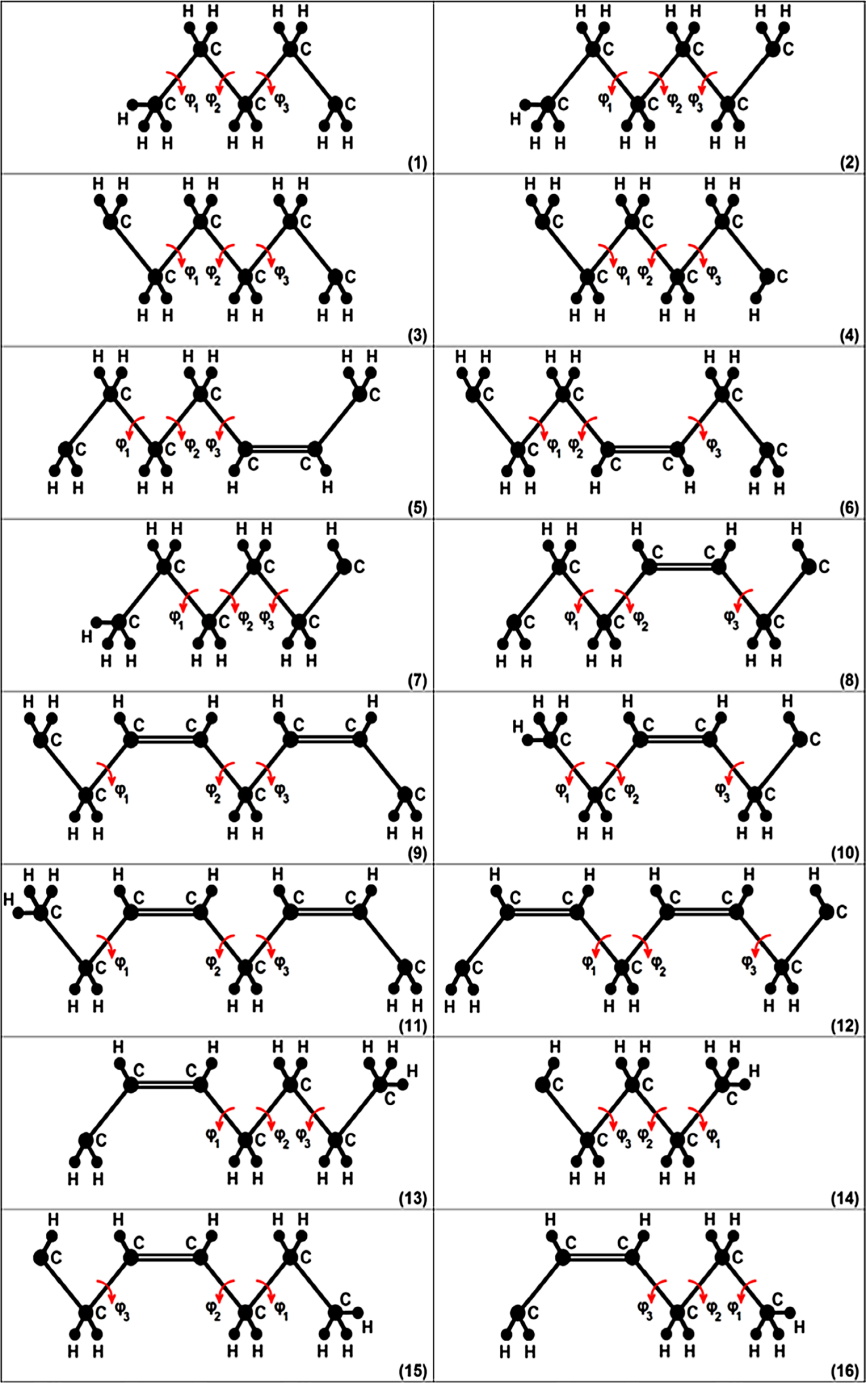
Sixteen structural units; to construct a linear hydrocarbon (n-alkane or n-alkene) chain of the structure, e.g., as in Fig. 2, that is typical for the biomembrane phospholipid chain structure (Fig. 1), and calculate the energy *U* according to expression (4), several of the presented units should be properly combined. Three variable torsions in each unit are marked by *red arrows*. The *number* at the bottom right of the unit is the unit’s number

To construct a chain of the above-mentioned structure and then calculate the energy *U* according to expression (4), several of the 16 structural units presented in Fig. 4 should be properly combined. Three variable torsions in each unit in Fig. 4 are marked by red arrows. It is seen from expression (4) that the structural units in a chain should be connected in such a way that each two consecutive (neighboring) units have two mutually variable torsions.

When calculating $U_m$ of each structural unit, the torsion energy, non-bonded energy and electrostatic energy from the force field were considered; bond lengths and bond angles were fixed at equilibrium values. Therefore bond length energy and bond angle energy were constant. In the calculations, multipliers of 1/2 and 1/3 were used for some energy items to exclude the possibility of double (or triple) summation of any energy items in the final expression (4). The multiplier 1/2 was used for the energy items which are strictly dependent on two variable torsions, and 1/3 was used for items strictly dependent on one variable torsion. The energy items that are strictly dependent on the three variable torsions of the considered structural unit were accounted without multipliers (i.e., a multiplier was equal to 1). It should be recorded that, at this step, it is possible to calculate the short-range energy *U* according to (4) only approximately; nevertheless, it is quite sufficient for the next step of the algorithm. An exact calculation of the energy *U* of each conformation is carried out after it has been generated—see below: it will be denoted as $U_{\rm units}$ in the final assessment $\overline{H}_{\omega }$ of the value $\langle H \rangle _{\Theta }$.

**Fig. 5:**
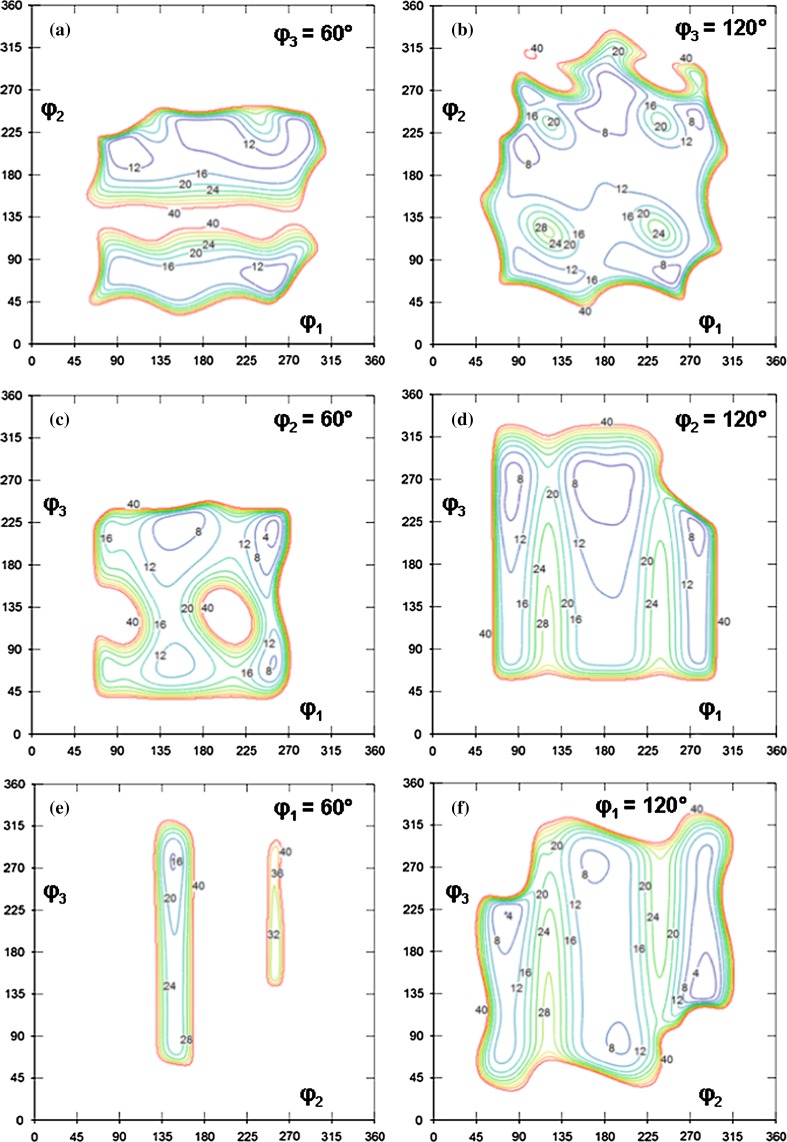
Six two-dimensional energy maps of structural unit 9 from Fig. 4. The value 0$^{\circ }$ of any torsions (angles $\varphi _1$, $\varphi _2$ and $\varphi _3$) corresponds to the eclipsed conformation. The numbers near equienergetic contours are energies (kJ/mol). The energy of each map is measured from the global energy minimum of structural unit 9

It is possible to demonstrate here an ’interdependence phenomenon’ of three consecutive variable torsions along the chain by the example of structural unit 9 from Fig. 4. Energy $U_9 (\varphi _1, \varphi _2, \varphi _3)$ of unit 9 was calculated (tabulated) and presented in Fig. 5 in the form of six two-dimensional energy maps containing equienergy contours, i.e., lines connecting the points of equal energies.

The variants presented in Fig. 5 contain

1. Equienergy contours for $(\varphi _1, \varphi _2)$ pair at two fixed values of $\varphi _3$ ($\varphi _3 = 60^{\circ }$, Fig. 5a, and $\varphi _3 = 120^{\circ }$, Fig. 5b),
2. Equienergy contours for $(\varphi _1, \varphi _3)$ pair at two fixed values of $\varphi _2$ ($\varphi _2 = 60^{\circ }$, Fig. 5c, and $\varphi _2 = 120^{\circ }$, Fig. 5d),
3. Equienergy contours for $(\varphi _2, \varphi _3)$ pair at two fixed values of $\varphi _1$ ($\varphi _1 = 60^{\circ }$, Fig. 5e, and $\varphi _1 = 120^{\circ }$, Fig. 5f).

Equienergy contours for a pair of torsions can, of course, be demonstrated at any fixed value of the third torsion angle (not only at 60$^{\circ }$ and 120$^{\circ }$). It is seen that map (a) of Fig. 5 is significantly different from map (b) of this figure; a large difference is observed for maps (c) and (d), maps (e) and (f), i.e., dependence of energy $U_9$ on torsion angles $\varphi _1, \varphi _2, \varphi _3$ is pronounced (in other words, interdependence exists between the three torsions).

To generate the density (3) for a given chain, a special technique was developed. Let $N_f = N-3$ for simplicity; then, we can substitute *U* from (3) by expression (4) and rewrite expression (3) in a different form:

5 $$\begin{aligned} p(\varphi _1,\ldots , \varphi _{N-1}) \approx \frac{ \prod \limits _{\gamma =1}^{N-3} \exp [-U_{m_{\gamma }}(\varphi _{\gamma },\varphi _{\gamma +1},\varphi _{\gamma +2})/k_BT]}{\int \limits _0^{2\pi }\ldots \int \limits _0^{2\pi } \prod \limits _{\gamma =1}^{N-3} \exp [-U_{m_{\gamma }} (\varphi _{\gamma },\varphi _{\gamma +1},\varphi _{\gamma +2})/k_BT] \cdot d\varphi _1\ldots d\varphi _{N-1}}. \end{aligned}$$

The energy $U_m (\varphi _{\gamma }, \varphi _{\gamma + 1}, \varphi _{\gamma + 2})$ of each structural unit *m* was tabulated with a step of $1^{\circ }$. Then $exp[- U_m(\varphi _{\gamma }, \varphi _{\gamma + 1}, \varphi _{\gamma + 2}) / k_BT]$ values under given *T* were calculated, and integrals

$$\begin{aligned} {\int \limits _0^{2\pi } \int \limits _0^{2\pi } \int \limits _0^{2\pi } \exp [- U_m(\varphi _{\gamma }, \varphi _{\gamma + 1}, \varphi _{\gamma + 2}) / k_BT] d \varphi _{\gamma } d \varphi _{\gamma + 1} d \varphi _{\gamma + 2}} \end{aligned}$$

were computed numerically. Then, the configurational space of torsion angles $\varphi _{\gamma },\varphi _{\gamma +1},\varphi _{\gamma +2}$ of each chain’s unit *m*, where $0\le \varphi _{\gamma }\le 2\pi , ~0\le \varphi _{\gamma +1}\le 2\pi , ~0\le \varphi _{\gamma +2}\le 2\pi$ (i.e., a ‘cube’), was divided numerically into 100$^3$ = 1,000,000 parallelepipeds (’states’) in such a way that they all have equal Boltzmann realization probabilities under given *T*. As a result, boundaries between the ’states’ along three directions (torsions) in angle units were calculated; to do that, a special mathematical algorithm was developed. The idea of the algorithm is as follows. At first, values of boundaries along $\varphi _{\gamma }$ axis were calculated using a recurrent relationship, to divide the ‘cube’ onto 100 quadratic ($2\pi \times 2\pi$) strata (‘layers’) having equal realization probabilities and, as a consequence, different widths. Then, each stratum, using a recurrent relationship, was numerically divided along the second axis, $\varphi _{\gamma +1}$, onto 100 ‘columns’ (‘rods’) in such a way that all ’columns’ have equal realization probabilities (and, because of this, different sizes); the values of boundaries between the ‘columns’ were obtained. Finally, each ‘column’ was similarly divided along the third axis, $\varphi _{\gamma +2}$, onto 100 equiprobable parallelepipeds (‘states’) and, hence, all sizes (edge lengths in angle units) of the parallelepipeds are not equal to each other. It is evident that with this method of splitting, the boundaries of parallelepipeds of each structural unit *m* gather in the areas where energy minima, i.e. the number of states (parallelepipeds) in the energy minima is much more than those around the maxima.

The calculated boundaries between 1,000,000 equiprobable parallelepipeds for each chain’s unit *m* are then used in MC simulations of the different hydrocarbon chains. The current chain conformation (a set of values of torsions along the chain) is generated randomly by selection of the stratum, column and parallelepiped numbers and then by selection of exact values of the torsions inside the chosen parallelepipeds. In doing so, the proper sequences of structural units of the given chain and the torsion numbers along the chain are obeyed.

The final assessment $\overline{H}_{\omega }$ of the value $\langle H \rangle _{\Theta }$ within the methodology can be obtained by

6 $$\begin{aligned} \overline{H}_{\omega } = \frac{\sum \limits _{\nu = 1}^\omega H( \varphi _1^\nu ,\ldots , \varphi _{N-1}^\nu ) \cdot \exp [ - U_{\rm units}(\varphi _1^\nu ,\ldots , \varphi _{N-1}^\nu ) / k_B T] \cdot W^\nu }{\sum \limits _{\nu = 1}^\omega \exp [ - U_{\rm units}(\varphi _1^\nu ,\ldots , \varphi _{N-1}^\nu ) / k_B T] \cdot W^\nu }. \end{aligned}$$

where

7 $$\begin{aligned} W^\nu = [(L_1)_{{m_1},{\lambda ^{\nu }_{1,1}}} (L_2)_{{m_1},{\lambda ^{\nu }_{2,1}}} (L_3)_{{m_1},{\lambda ^{\nu }_{3,1}}} ] \cdot \prod \limits _{\gamma = 2}^{N - 3} (L_3)_{{m_{\gamma }},{\lambda ^{\nu }_{3,{\gamma }}}} \end{aligned}$$

Here, $(L_1)_{{m_\gamma },{\lambda ^{\nu }_{1,\gamma }}}$, $(L_2)_{{m_\gamma },{\lambda ^{\nu }_{2,\gamma }}}$ and $(L_3)_{{m_\gamma },{\lambda ^{\nu }_{3,\gamma }}}$   are sizes (edge lengths in angle units) of a parallelepiped randomly chosen for the $\nu$-th conformation of the chain in the molecular unit number $\gamma$ of type *m*; $\lambda ^{\nu }_{1,\gamma }$, $\lambda ^{\nu }_{2,\gamma }$ and $\lambda ^{\nu }_{3,\gamma }$ are random numbers of the three edges of the chosen parallelepiped; $U_{\rm units}(\varphi _1^\nu ,\ldots , \varphi _{N-1}^\nu )$ is the short-range interactions energy of the generated $\nu$-th chain conformation, properly calculated using all terms of the force field: all bond length and angle energies, all torsion (dihedral) angle energies, out-of-plane energies, Urey-Bradley terms, and non-bonded and electrostatic interactions energies for such pairs of atoms which are included into the sequence of structural units of the given chain. Since all values of the torsions in the generated conformation are already known, the short-range energy $U_{\rm units}$ is calculated correctly [in contrast to the energy *U* calculated approximately from expression (4)].

Thus, in the assessment (6), the probability of generation of each chain conformation and probability of its realization are calculated, and hence we obtain $\overline{H}_{\omega }\xrightarrow {\omega \rightarrow \infty }\langle H \rangle _{\Theta }$. To calculate average characteristics, approximately 10$^{12}$ conformations of each chain were generated in the present work.

## Results and discussion

Average distances between terminal carbon atoms of the chains (end-to-end distances) considered in the unperturbed state, $\langle h \rangle _{\Theta }$, and those of PC lipid chains in liquid crystalline bilayers, $\langle h \rangle _{bil}$, were calculated. The obtained data are presented in Table 8.

Table 8 shows that $\langle h \rangle _{\Theta }$ values are somewhat less than $\langle h \rangle _{bil}$. This is because only one hydrocarbon chain terminus in the lipid molecule is free in space; the other one is chemically linked to the head group. Due to interactions of all lipid molecules with their neighbors and water molecules the lipids’ head groups are arranged in the vicinity to each other. Therefore, possibilities of rotations around several C–C bonds adjoining the head groups are more restricted than those for the C–C bonds of the opposite end of the chain, so the chain region near the head groups is more stretched, in contrast to the unperturbed chain in which both ends are free.

**Table 8:** Average end-to-end distances, $\langle h \rangle _{\Theta }$ for unperturbed hydrocarbon chains and $\langle h \rangle _{bil}$ for the acyl chains in liquid crystalline phosphatidylcholine (PC) bilayers obtained by computer simulations of the present work; $T = 303$ K

Hydrocarbon chain, MC simulation	$\langle h \rangle _{\Theta }$ ($\pm \Delta _1$)^a^, [nm] Unperturbed state MC simulation	Acyl chain, MD simulation	$\langle h \rangle _{\rm bil}$ ($\pm \Delta _2$)^b^, [nm] 16:0/... PC bilayer MD simulation	$\langle h \rangle _{\rm bil}$ ($\pm \Delta _2$)^b^, [nm] 18:0/... PC bilayer MD simulation	Relat. diff. expression (8)) $\delta$, $\%$
*alk*-16:0	1.395 (±0.001)	16:0	1.503 – 1.520^c^ (±0.001)		7.2 – 8.2^e^
*alk*-18:0	1.528 (±0.001)	18:0		1.665 – 1.699^d^ (±0.001)	8.3 – 10.1^f^
*alk*-18:1	1.374 (±0.001)	18:1	1.532 (±0.001)	1.531 (±0.001)	10.3; 10.3^g^
*alk*-18:2	1.293 (±0.001)	18:2	1.478 (±0.001)	1.491 (±0.001)	12.5; 13.3^g^
*alk*-18:3	1.261 (±0.001)	18:3	1.447 (±0.001)	1.446 (±0.001)	12.8; 12.8^g^
*alk*-18:4	1.194 (±0.003)	18:4	1.398 (±0.001)	1.402 (±0.001)	14.6; 14.8^g^
*alk*-18:5	1.165 (±0.008)	18:5	1.372 (±0.001)	1.373 (±0.001)	15.1; 15.1^g^
*alk*-20:4	1.290 (±0.002)	20:4	1.544 (±0.001)	1.538 (±0.001)	16.5; 16.1^g^
*alk*-20:5	1.254 (±0.001)	20:5	1.497 (±0.001)	1.509 (±0.001)	16.2; 16.9^g^
*alk*-22:6	1.328 (±0.005)	22:6	1.624 (±0.001)	1.645 (±0.001)	18.2; 19.3^g^

^a^ stat. error $\Delta _1$ evaluated from the variance of $\sim$ 10$^{12}$ conformations

^b^ stat. error $\Delta _2$ for 20–100 ns evaluated from the variance of 10 ns block averages

^c^ range of $\langle h \rangle _{\rm bil}$ for 16:0 acyl chain in eight mixed-chain 16:0/... PC bilayers

^d^ range of $\langle h \rangle _{\rm bil}$ for 18:0 acyl chain in eight mixed-chain 18:0/... PC bilayers

^e^ range of $\delta$ values for 16:0 acyl chain in eight mixed-chain 16:0/... PC bilayers

^f^ range of $\delta$ values for 18:0 acyl chain in eight mixed-chain 18:0/... PC bilayers

^g^ values of $\delta$ for this unsaturated acyl chain in 16:0/... PC and 18:0/... PC bilayers, respectively

To compare the results quantitatively, the relative (in percentage) difference $\delta$ between values $\langle h \rangle _{\rm bil}$ and $\langle h \rangle _{\Theta }$ for each chain was calculated:

8 $$\begin{aligned} \delta = [(\langle h \rangle _{\rm bil} - \langle h \rangle _{\Theta })/\langle h \rangle _{\rm bil}] \cdot 100. \end{aligned}$$

The values of $\delta$ are also presented in Table 8. The calculations showed that the relative difference $\delta$ between both states increases as the number of carbons and/or number of double bonds in the chain increase; $\delta$ is approximately equal to 8–10% (or $\sim$9%) for saturated 16:0 and 18:0 chains; to 10–15% for unsaturated chains with *N* = 18, *d* = 1–5; to 16–17% for PU chains with *N* = 20, *d* = 4 – 5 and maximum $\sim$19% for 22:6(n-3)*cis* chain.

In this connection, a remark should be made. It is possible to divide interactions in the bilayers into three parts: intramolecular short-range, intramolecular long-range interactions (Flory 1969) of the chains, and intermolecular interactions between the chains and the neighboring chains (and PC head groups). Unperturbed hydrocarbon chain properties are fully defined by the short-range interaction energy (Flory 1969). Therefore, the value of $\delta$ can be considered as an assessment of the influence of the long-range interactions inside the chain and interactions with the neighboring chains and PC head groups of the lipid bilayer on the distance $\langle h \rangle _{\rm bil}$, compared with the influence of only short-range interactions inside the chain on the $\langle h \rangle _{\Theta }$.

While conformations of the unperturbed chains are not the same as those in liquid crystalline bilayers, the relative difference $\delta$ between the end-to-end distances $\langle h \rangle _{bil}$ and $\langle h \rangle _{\Theta }$ of the considered typical acyls was found to be comparatively moderate; it is approximately equal to 9–19%.

Therefore, such properties of the listed FA chains of phospholipids in bilayers, as $\langle h \rangle _{\rm bil}$ are significantly determined by the short-range interactions in the chains (indeed, it is determined approximately by 81–91%). There is good reason to believe that other geometrical properties of these chains are also determined mainly by their short-range interactions. We mention that the considered saturated and unsaturated chains are widespread, typical constituents of phospholipids, so the obtained result seems to be valuable for biomembranes. In other words, the $\delta$ value seems to be of the same order of magnitude for most of different membrane hydrocarbon chains with methylene-interrupted *cis* double bonds.

It should be also pointed out, that a relationship between $\langle h \rangle _{\Theta }$ and the number *d* of double bonds for the unperturbed hydrocarbon chains with constant number *N* of carbon atoms is the same as $\langle h \rangle _{\rm bil}$ for acyl chains of PC molecules in bilayers: both values decrease as *d* increases (Table 8). In other words, the trends in changes of $\langle h \rangle _{\Theta }$ and $\langle h \rangle _{\rm bil}$ in the order 18:0 $\rightarrow$ 18:1 $\rightarrow$ 18:2 $\rightarrow$ 18:3 $\rightarrow$ 18:4 $\rightarrow$ 18:5 are the same; the trends in the order 20:4 $\rightarrow$ 20:5 are also the same.

This decrease is obviously caused mainly by extension of the chain segment with double bonds, in which energy minima are wide and correspond to various collapsed chain conformations, and by shortening of chain’s saturated segments in which the main energy minimum corresponds to the extended chain conformations (Flory 1969).

**Fig. 6:**
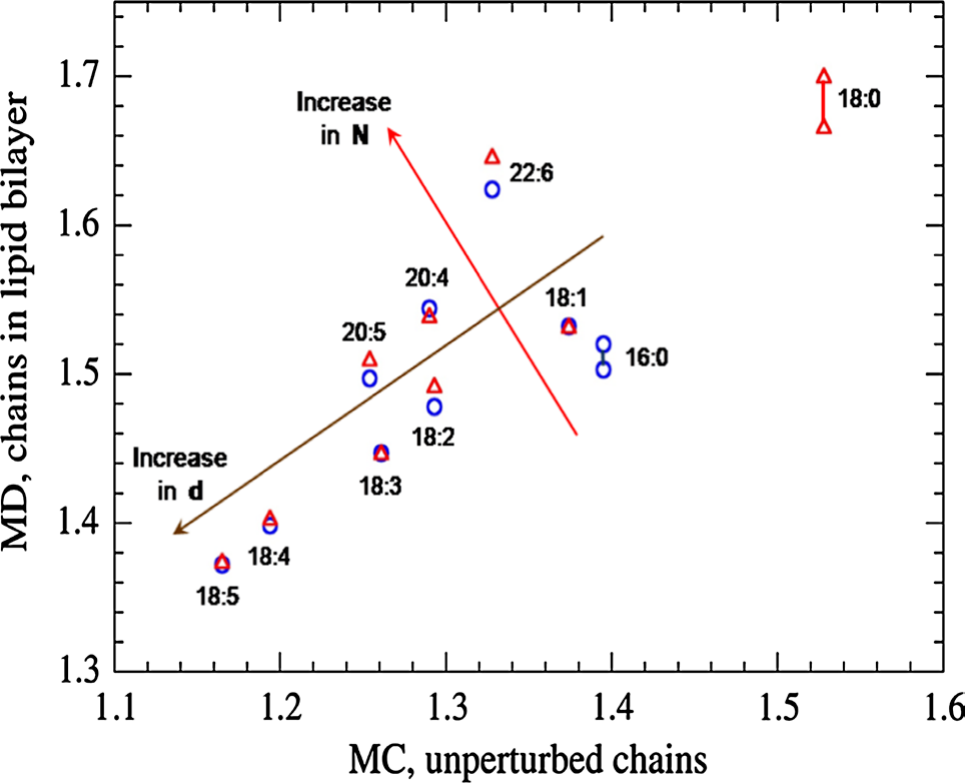
Average end-to-end distances $\langle h \rangle _{\Theta }$, [nm], for unperturbed hydrocarbon chains obtained by Monte Carlo (MC) simulations, and $\langle h \rangle _{\rm bil}$, [nm], for the same acyl chains in liquid crystalline phosphatidylcholine (PC) bilayers obtained by molecular dynamics (MD) simulations (triangles for the marked chains in 18:0/... PC bilayers, circles for the marked chains in 16:0/... PC bilayers). The ranges for saturated acyl chains 16:0 and 18:0 are obtained for eight appropriate mixed-chain PC bilayers. Computer simulations of the present work, $T = 303$ K. *Arrows* show qualitatively trends in $\langle h \rangle _{\Theta }$ and $\langle h \rangle _{\rm bil}$. To compare the obtained trends, the same names used here both for acyls (ordinate axis) and hydrocarbon chains (abscissa axis)

It is possible to consider also the trends of both discussed values of $\langle h \rangle$ in the order 18:4 $\rightarrow$ 20:4 and the trends in the order 18:5 $\rightarrow$ 20:5, i.e., relationships between $\langle h \rangle _{\Theta }$, $\langle h \rangle _{\rm bil}$ and number *N* of carbon atoms under constant number *d* of double bonds. Table 8 shows that they are similar to each other, respectively. The discussed trends are shown schematically in Fig. 6. Of course, more rigorous treatment requires consideration also of the value of the parameter *j* in all trends, i.e., the third structure parameter of the chain in the expression $N:d(n-j)cis$ that means the positions of double bonds along the chain. In particular, *j* differs for the considered chains 18:4 and 20:4, 20:4 and 20:5, and for some chains with $N = 18$.

To our knowledge, a quantitative assessment of the difference between properties of the two chain states (in particular, the difference in $\langle h \rangle$ values) was obtained for the first time in the present work, while a qualitative similarity of chain properties in the two states was discussed in the literature before. For instance, it has been concluded (Rabinovich et al. 2003) that the bond-order parameters and orientation distribution characteristics of the chains in the lipid monolayer and bilayer ‘liquid’ regions, as found in experiments and in MD computer simulation models (Rabinovich et al. 1999a, b, 2000; Rabinovich and Balabaev 2001), are qualitatively similar to the intramolecular order parameters and the intramolecular bond orientation distributions in single unperturbed (Flory 1969) unsaturated hydrocarbon chains previously investigated with MC simulations (Rabinovich and Ripatti 1999, 2000). Therefore, the behavior of the acyl chains in the liquid region of lipid bilayers (somewhat remote from the membrane–water interface) is dominated by the intramolecular short-range interactions. The long-range interactions of the segments of the lipids in this region of the bilayer and the interactions with the bilayer–water interface may be considered as a disturbance: the intermolecular interactions are largely used to orient the lipid molecules in the direction of the membrane normal.

Thus, from the two above-mentioned facts (about a comparatively moderate quantitative difference in the $\langle h \rangle _{\rm bil}$ and $\langle h \rangle _{\Theta }$ values of the chains and the similarity of their trends), a common conclusion can be made: to treat and analyse a number of processes in biological membranes (e.g., changes in FA composition caused by the environmental changes such as temperature and pressure), it is possible to use, at least as the first approximation, the relationships between structure and properties obtained for the unperturbed hydrocarbon chains. This seems not unreasonable: biomembranes are known to contain a wide variety of FA chains; the available ‘bilayer’ relations between their structure and property are incomplete and insufficient for the analysis, whereas the properties of the unperturbed chains and corresponding ‘structure–property’ relationships have been already studied for tens of variants (see, e.g., Zhurkin and Rabinovich 2015).

## Conclusions

The average characteristics of hydrocarbon chains calculated in the unperturbed state (which is fully defined by short-range interaction energies) make it possible to estimate the influence of additional energy components on the state of these chains, if they are under other conditions or located in other systems. For the same temperature and force field parameters, it is acceptable to use any characteristic as a criterion. The average end-to-end distance of the chains was chosen as such a criterion in the computer simulations carried out in this work. The relationships between structure and the average end-to-end distances obtained for the considered unperturbed chains were shown to be qualitatively similar to those of lipid chains in bilayers. Such data for the majority of possible lipid acyls in bilayers (as a rule, it is several tens of chains or more) are yet unknown because MD simulations of various lipid bilayers are very time-consuming. On this basis, it is reasonable to investigate the unperturbed hydrocarbon chains instead. As a first approximation of the desired ‘structure–property’ relationships for the lipid chains in bilayers, the corresponding relationships for the unperturbed chains can be used.
